# Designs used in published therapeutic studies of rare superficial vascular anomalies: a systematic literature search

**DOI:** 10.1186/s12874-023-02017-0

**Published:** 2023-08-30

**Authors:** Aude Allemang-Trivalle, Sophie Leducq, Annabel Maruani, Bruno Giraudeau

**Affiliations:** 1 Université de Tours, Université de Nantes, INSERM, SPHERE U1246, Tours, France; 2 INSERM CIC1415, CHRU de Tours, Tours, France; 3 Department of Dermatology, Reference Center for Genodermatoses and Rare Skin Diseases (Maladies Génétiques rares à Expression Cutanée–Tours), CHRU de Tours, Tours, France

**Keywords:** Research Design, Randomized controlled trials, Vascular malformations, Rare Diseases

## Abstract

**Background:**

Rare superficial vascular anomalies represent a wide range of diseases. Their management is difficult given the broad spectrum and the lack of clinical trials assessing treatment efficacy. A randomized clinical trial of vascular anomalies is difficult because of the rarity of the diseases and is enhanced by the population of interest often being children. Therefore, suitable designs are needed. We conducted a methodological systematic literature search to identify designs implemented for investigating the treatment of rare superficial vascular anomalies.

**Methods:**

We conducted a literature search on January 25, 2021, of the PubMed, Cochrane Central Register of Controlled Trials (CENTRAL), Embase, ClinicalTrials.gov and European Union Clinical Trials Register databases. This systematic methodological literature search was registered at the Prospective Register of Systematic Reviews (PROSPERO: CRD42021232449). Randomized and non-randomized studies were included if they met the following criteria: were prospective studies of rare superficial vascular anomaly therapies, dealt with humans (adults and children) and were published in English from 2000. We excluded case reports/case series reporting fewer than 10 patients, reviews, retrospective studies, animal studies, studies of systemic or common vascular anomalies and non-therapeutic studies. We did not assess risk of bias in the included studies because our review was a methodological one focused on the design used. The review provided a descriptive analysis of relevant features of eligible research studies.

**Results:**

From 2046 articles identified, we included 97 studies (62 reports and 35 ongoing studies): 25 randomized controlled studies, 7 non-randomized comparative studies, 64 prospective cohorts and 1 case series. Among the 32 comparative studies included, 21 used a parallel-group design. The 11 other studies used different designs such as cross-over, randomized placebo phase, delayed-start, within-person, or challenge–dechallenge–rechallenge or used a historical control group or an observational run-in period.

**Conclusions:**

Our systematic literature search highlights the lack of randomized control trials in superficial vascular anomalies due to the rarity of patients and their heterogeneity. New designs are emerging and can overcome the limitations of testing treatments in parallel groups.

**Supplementary Information:**

The online version contains supplementary material available at 10.1186/s12874-023-02017-0.

## Background

Vascular anomalies (VAs) represent a large panel of diseases and are classified according to their clinical, biological, radiological, pathological and molecular characteristics by the International Society for the Study of Vascular Anomalies (ISSVA) as vascular tumors (VTs), which might be benign, borderline or malignant, or vascular malformations (VMs) [1]. VTs are characterized by endothelial-cell proliferation or hyperplasia, whereas VMs result from a defect in embryonic vasculogenesis that might be linked to somatic or germinal gene mutations. VMs are classified according to the vessels involved (i.e., capillary VMs [port-wine stains], venous VMs, lymphatic VMs or arteriovenous VMs). They are considered “simple” when one type of vessel is involved and combined or syndromic if associated with other malformations [2–4]. This classification does not take into account the prevalence of the conditions, which might be common (port-wine stains, infantile hemangiomas) or rare. Rare vascular anomalies often are chronic conditions: in Europe, a disease affecting fewer than 1 in 2,000 people is considered a rare disease [5], whereas in the United States, a rare disease is defined as a condition that affects fewer than 200,000 people [6]. The natural history of most rare VAs is progressive worsening without therapeutic intervention.

Treatment of rare VAs includes different therapeutic modalities (i.e., physiotherapy, interventional radiology [sclerotherapy/embolization], surgery, interventions with device such as lasers, and drugs [anticoagulants, mammalian target of rapamycin inhibitors], including targeted therapeutic ones such as selective inhibitor of the phosphoinositol-3-kinase for CLOVES syndrome) [7–10]. Often, treatments are started in childhood. The management of rare VAs is based on a personalized approach that considers the patient’s goals for treatment and usually requires multidisciplinary consultations. There are no validated guidelines for treatment of rare VAs [11]. Indeed, recommendations are difficult to develop given the broad spectrum and insufficient number of prospective clinical studies to prove the efficacy of treatment [7, 9, 12].

Randomized controlled trials (RCTs) provide gold-standard evidence to guide clinical practice. However, trials for rare diseases have recruitment issues and require tailored designs. Regarding rare VAs, the design of clinical trials is difficult because of the cumulative difficulty of the rarity of diseases, the heterogeneity of conditions and often that the population of interest is children, which, above recruitment issues, raises specific ethical issues (consent of both parents, acceptance by the child, limited number of samples and invasive exams) [13, 14]. To overcome these methodological and ethical problems, alternative designs have emerged but are still little used [15].

The aim of this study was to investigate the study designs used in therapeutic clinical studies of rare VAs by a systematic literature search.

## Methods

### Registration and protocol

This study was designed as a systematic methodological literature. It was registered at the Prospective Register of Systematic Reviews (PROSPERO: CRD42021232449).

### Eligibility criteria

Reports of randomized and non-randomized studies were included if they met the following criteria: were prospective studies of rare superficial VA therapies, dealt with humans (adults and children) and were published in English from 2000. The different groups of VAs included were simple lymphatic malformations, simple venous malformations, slow-flow combined malformations including syndromic forms, arteriovenous malformations and rare vascular tumors. We included ongoing studies (i.e., studies, comparative or not, whose results were not published at the time of the electronic research on January 25, 2021). We excluded case reports/case series reporting fewer than 10 patients, reviews, retrospective studies, animal studies, studies of systemic or common VAs (prevalence greater than 1 in 2,000 people) and non-therapeutic studies. There was no minimum number of patients for clinical trials. To differentiate the prospective cohorts from case series, we followed the definition proposed by Dekkers et al. [16]: “in a cohort study, patients are sampled on the basis of exposure and are followed over time, and the occurrence of outcomes is assessed.” In a case series, patients can be sampled according to both the specific outcome and specific exposure or only a specific outcome. To distinguish cohort studies from case series, we mainly considered the participant selection and sampling parameter (Is it linked to the outcome? to a specific exposure?) and to the presence of a follow-up period during which the outcome is assessed (cohort study criteria) [17]. Two authors reviewed the full text of each study, with blinding, to label them. Discrepancies were resolved by discussion between these two authors. Many studies that could have been initially defined as case series were reclassified as cohort studies.

### Search strategy

The electronic search conducted on January 25, 2021, involved the databases PubMed, Embase (on Embase.com), and Cochrane Central Register of Controlled Trials (CENTRAL) and the registers ClinicalTrials.gov and European Union Clinical Trials Register (EUCTR). The search terms used can be found in the supplementary materials and methods file.

### Selection process

According to the pre-defined criteria, two authors (AAT and SL) independently selected reports based on the title abstracts. Any discrepancies were resolved by the senior authors (AM and BG). The same two authors then examined the full texts of the selected reports. They excluded duplicate publications, general reviews, systematic reviews or reports with insufficient information as full text not accessible. Publication duplicates were detected by using first Zotero software and then Airtable. Duplicates between registers and publications were manually detected and resolved directly on Airtable.

### Data collection process

For each selected study, two reviewers independently followed a standard template for data extraction. Any disagreements were resolved by discussion and if necessary by a senior author. Data were extracted to an Airtable spreadsheet (https://airtable.com/) and included publication metrics (name of the first author, journal, publication year, source and article type), study recruitment characteristics (continent and number of centres), study design (study type, randomization, trial design, design justification by the authors, blinding and important changes after trial beginning), study groups (number of groups, experimental treatment and control), primary outcome, characteristics of patients (type of VAs using the ISSVA classification system, age category), planned sample size, number of patients included and funding sources. Intervention type in the experimental group were extracted and classified as follows: parenteral drugs, enteral drugs, topical drugs, interventional radiology (i.e., image-guided minimally invasive treatment such as embolization or percutaneous sclerosis) and physiotherapy. Figure S1 (supplementary material) represents the designs used in comparative studies included in the final analysis.

### Data analysis

We did not assess risk of bias in the included studies. The review provided a descriptive analysis of relevant features of eligible research studies, focusing on designs used. The characteristics of each included clinical trial were summarized in tables using descriptive statistics. Categorical variables are presented as counts and proportions (n, %). Quantitative variables are presented as median and interquartile range (median [Q1-Q3]). To statistically analyze categorical variables, we used the chi-square test. To statistically analyze quantitative variables, we used the non-parametric Mann-Whitney U test. R-4.2.2 software was used for statistical analyses.

## Results

**Fig. 1:**
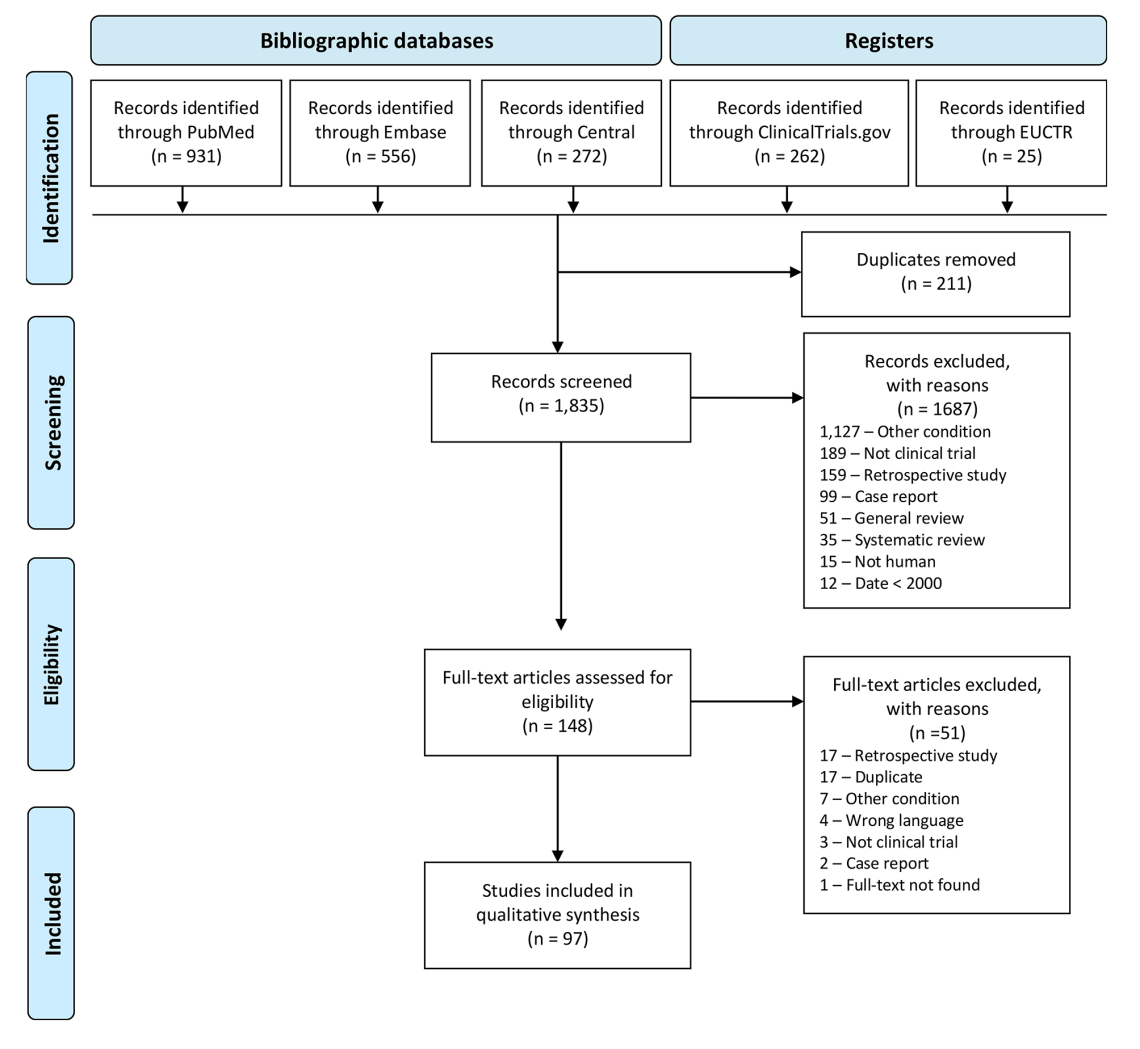
Flow of articles in the review

In the initial database search, we identified 2 046 reports and finally included 97 studies (62 reports and 35 ongoing studies): 25 RCTs, 7 non-randomized comparative studies, 64 prospective cohort studies and 1 case series. Figure 1 presents the flow of articles in the review.

### Characteristics of the included studies

Table 1 shows the epidemiology and reporting characteristics of the 97 included studies. Twenty studies that could have been initially defined as case series were reclassified as cohort studies [18–37].

**Table 1:** Description of studies included in the final analysis

ALL STUDIES						
		**TOTAL** **N = 97**	**REPORTS** **N = 62**		**ONGOING STUDIES** **N = 35**	
SOURCE	Publication year, n (%)					
	2002–2010	20 (20.6)	17 (27.4)		3 (8.6)	
	2011–2015	28 (28.9)	23 (37.1)		5 (14.3)	
	2016–2020	49 (50.5)	22 (35.5)		27 (77.1)	
	Source, n (%)					
	Bibliographic databases	70 (72.2)	62 (100.0)		8 (22.9)	
	Registers	27 (27.8)	0		27 (77.1)	
SETTING	International study, n (%)					
	Yes	9 (9.3)	3 (4.8)		6 (17.1)	
	No	87 (89.7)	58 (93.6)		29 (82.9)	
	Unclear	1 (1.0)	1 (1.6)		0	
	Continent of study recruitment, n (%) ^a^					
	North America	15 (15.5)	6 (9.7)		9 (25.7)	
	South America	1 (1.0)	1 (1.6)		0	
	Europe	26 (26.8)	12 (19.4)		14 (40.0)	
	Asia	53 (54.6)	39 (62.9)		14(40.0)	
	Africa	4 (4.1)	4 (6.5)		0	
	Oceania	1 (1.0)	0		1 (2.9)	
	Unclear	1 (1.0)	1 (1.6)		0	
	Number of centres, n (%)					
	1	72 (74.2)	53 (85.5)		19 (54.3)	
	2–5	14 (14.4)	5 (8.1)		9 (25.7)	
	> 5	9 (9.3)	2 (3.2)		7 (20.0)	
	Unclear	2 (2.1)	2 (3.2)		0	
FUNDING	Funding body, n (%)					
	Public	43 (44.3)	13 (21.0)		30 (85.7)	
	Private	6 (6.2)	1 (1.6)		5 (14.3)	
	Both private and public	1 (1.0)	1 (1.6)		0	
	No specific funding	4 (4.1)	4 (6.5)		0	
	Not reported	43 (44.3)	43 (69.4)		0	
DESIGN	Study type, n (%)					
	Comparative	32 (33.0)	15 (24.2)		17 (48.6)	
	Prospective cohort	64 (66.0)	46 (74.2)		18 (51.4)	
	Case series	1 (1.0)	1 (1.6)		0	
PARTICIPANTS	Study population type, n (%)					
	Children	28 (28.9)	17 (27.4)		11 (31.4)	
	Adults	5 (5.1)	0		5 (14.3)	
	Both	63 (65.0)	44 (71.0)		19 (54.3)	
	Unclear	1 (1.0)	1 (1.6)		0	
	Included participant diseases, n (%) ^a^					
	Simple lymphatic malformations	51 (52.6)	37 (59.7)		14 (40.0)	
	Simple venous malformations	52 (53.6)	37 (59.7)		15 (42.9)	
	Slow flow combined malformations including syndromic forms	21 (21.7)	10 (16.1)		11 (31.4)	
	Arteriovenous malformations	14 (14.4)	7 (11.3)		7 (20.0)	
	Rare vascular tumors	18 (18.6)	9 (14.5)		9 (25.7)	
	Several diseases	27 (27.8)	18 (29.0)		9 (25.7)	
		**TOTAL** **N = 97**	**STUDY STATUS**		**STUDY OBJECTIVE**	
			**REPORTS** **N = 62**	**ONGOING STUDIES** **N = 35**	**COMPARATIVE STUDIES** **N = 32**	**NON-COMPARATIVE STUDIES** **N = 65**
INTERVENTION	Intervention type in the experimental group, n (%) ^a^					
	Parenteral drugs	3 (3.1)	2 (3.2)	1 (2.9)	2 (6.3)	1 (1.5)
	Enteral drugs	25 (25.8)	6 (9.7)	19 (54.3)	10 (31.3)	15 (23.1)
	Topical drugs	5 (5.2)	0	5 (14.3)	3 (9.4)	2 (3.1)
	Interventional radiology	61 (62.9)	52 (83.9)	9 (25.7)	15 (46.9)	46 (70.8)
	Physiotherapy	3 (3.1)	2 (3.2)	1 (2.9)	2 (6.3)	1 (1.5)
	Surgery	0	0	0	0	0
	Combined treatment	14 (14.4)	10 (16.1)	4 (11.4)	0	0
SAMPLE SIZE	Planned sample size, n (%)					
	≤ 10^b^	3 (3.1)	1 (1.6)	2 (5.7)	0	3 (4.6)
	11–24	6 (6.2)	1 (1.6)	5 (14.3)	3 (9.4)	3 (4.6)
	25–49	12 (12.4)	1 (1.6)	11 (31.4)	6 (18.8)	6 (9.2)
	50–99	12 (12.4)	2 (3.2)	10 (28.6)	6 (18.8)	6 (9.2)
	≥ 100	7 (7.2)	1 (1.6)	6 (17.1)	4 (12.5)	3 (4.6)
	Not planned	57 (58.8)	56 (90.3)	1 (2.9)	13 (40.6)	44 (67.7)
	Number of patients included					
	Median	27.5	27.5	NA	39.0	20.0
	[Q1; Q3]	[15.0 ; 43.8]	[15.0 ; 43.8]	NA	[28.0 ; 89.5]	[13.5 ; 43.0]
	Number of patients included, n (%)					
	≤ 10	5 (5.2)	5 (8.1)	NA	0	5 (7.7)
	11–24	24 (24.7)	24 (38.7)	NA	2 (6.3)	22 (33.8)
	25–49	18 (18.6)	18 (29.0)	NA	8 (25.0)	10 (15.4)
	50–99	9 (9.3)	9 (14.5)	NA	2 (6.3)	7 (10.8)
	≥ 100	6 (6.2)	6 (9.7)	NA	3 (9.4)	3 (4.6)
	NA	35 (36.1)	35 (36.1)	NA	17 (53.1)	18 (27.7)

^a^Not exclusive

^b^Case reports/case series reporting fewer than 10 patients were excluded

Abbreviations: NA, not applicable; Q1, first quartile; Q3, third quartile

#### Patient characteristics

Most of the included studies involved heterogeneous patients. First, about two thirds of studies included both children and adults [7, 19–21, 23–27, 29–34, 36, 38–84]. Second, nearly one third were of several diseases [7, 22, 27, 29, 30, 33, 38, 40, 45, 50, 52, 54, 55, 57, 58, 60, 64, 65, 67, 68, 74, 77, 79, 84–86]. Most studies were of lymphatic malformations (LMs) and/or venous malformations [7, 18–34, 36–46, 48, 50–62, 64–68, 71–80, 82–104], followed by rare VTs [7, 27, 29, 35, 40, 52, 55, 60, 63, 64, 67, 68, 105–110], arteriovenous malformations [29, 38, 45, 50, 52, 55, 58, 67–70, 84, 111, 112], and slow-flow combined malformations or syndromic malformations [7, 22, 38, 45, 47, 49, 50, 52, 54, 57, 58, 64, 65, 68, 74, 77, 79, 81, 85, 86].

#### Experimental treatments

Overall, whatever the study type (comparative or non-comparative, randomized or non-randomized), the most evaluated experimental treatment was interventional radiology [18, 19, 21–27, 29, 30, 32–34, 36–42, 44–46, 50–57, 59–62, 66, 67, 72, 73, 75, 76, 78–80, 84, 87–94, 97, 98, 102, 104, 111–113] followed by enteral drugs [7, 20, 31, 47, 49, 63–65, 68–70, 74, 77, 81, 82, 85, 96, 99–101, 107–110]. total of 14 studies offered a combination of treatments in the experimental arm. For 10, it was a combination of two interventional radiology treatments [19, 32, 36, 39, 42, 44, 56, 59, 83, 91]. Three studies investigated the combination of two enteral drugs [47, 63, 100] and one study combined sclerotherapy and surgery [66]. Thus, many included studies used interventional radiology to treat LMs [19, 21–27, 29, 30, 33, 37–40, 42, 44, 45, 50–52, 54, 55, 57, 60, 75, 84, 97, 98, 102, 104, 113] (32 studies) and venous malformations [18, 22, 27, 29, 30, 32–34, 36, 38, 41, 45, 46, 50, 52–57, 59, 61, 62, 66, 67, 72, 73, 76, 78–80, 84, 87–94] (40 studies).

#### Planned and achieved sample size

Most studies (n = 57, 58.8%) did not report a planned sample size [18, 19, 21–30, 32–37, 39–41, 44–46, 51–57, 60–62, 66, 67, 72, 73, 75, 76, 78–80, 83, 84, 89, 91–94, 96–98, 100, 102, 104, 113]. A higher proportion of comparative studies [43, 48, 49, 59, 63, 65, 71, 74, 81, 85, 86, 88, 90, 99, 103, 105, 107, 109, 110] than non-comparative studies [7, 20, 31, 38, 42, 47, 50, 58, 64, 68–70, 77, 82, 87, 101, 106, 108, 111, 112] had a planned sample size (n = 19, 59.4% vs. n = 21, 32.3%, p = 0.011). For more than half the studies, the median (Q1-Q3) planned sample size was 38 (25–61) patients per study. In the 62 reports, the median (Q1-Q3) number of included patients was 28 (15–44) Comparative studies are significantly larger than non-comparative studies (median [Q1-Q3] sample size 39 [28–90] vs. 20 [14–43], p = 0.008). Table 1 presents the intervention and sample size description of studies included in the final analysis.

### Methodology of the studies

#### Comparative study design (32 studies)

Many of the comparative studies (n = 21, 65.6%) used a classical design with parallel groups [41, 44, 57, 59, 60, 63, 67, 72, 80, 83, 86, 88–91, 97, 99, 105, 107, 109, 110]. Table 2 presents the methodological data for comparative studies included in the final analysis. The 11 other studies used different designs: cross-over [48, 65], randomized placebo phase [43, 85], delayed-start [49, 103], within-person [71], challenge–dechallenge–rechallenge [74], and observational run-in period [81] or used a historical control group [66, 73]. Table 3 reports the characteristics of these studies. Two studies had two stages: a pilot phase with a small sample size followed by a larger comparative trial [43, 88].

**Table 2:** Methodological data for comparative studies included in the final analysis

COMPARATIVE STUDIES				
		**TOTAL** **N = 32**	**RANDOMIZED** **N = 25**	**NON-RANDOMIZED** **N = 7**
DESIGN	Trial design, n (%)			
	Parallel groups	21 (65.6)	19 (76.0)	2 (28.6)
	Cross-over	2 (6.3)	1 (4.0)	1 (14.3)
	Randomized placebo phase	2 (6.3)	2 (8.0)	0
	Within-person	1 (3.1)	1 (4.0)	0
	Delayed-start design	2 (6.3)	2 (8.0)	0
	Observational run-in period	1 (3.1)	0	1 (14.3)
	Challenge–dechallenge–rechallenge	1 (3.1)	0	1 (14.3)
	Use of a historical control group	2 (6.3)	0	2 (28.6)
	Studies reported design justifications, n (%)*			
	Yes	6 (18.8)	4 (16.0)	2 (28.6)
	No	26 (81.3)	21 (84.0)	5 (71.4)
	Blinding, n (%)			
	Participants only	2 (6.3)	2 (8.0)	0
	Participants and care provider	4 (12.5)	4 (16.0)	0
	Participants, care provider and outcome assessor	3 (9.4)	3 (12.0)	0
	Outcome assessor only	4 (12.5)	4 (16.0)	0
	Participants and outcome assessor	1 (3.1)	1 (4.0)	0
	No blinding	10 (31.3)	7 (28.0)	3 (42.9)
	Not specified	8 (25.0)	4 (16.0)	4 (57.1)
TREATMENT GROUPS	Number of groups, n (%)			
	1	8 (25.0)	8 (32.0)	NA
	2	15 (46.9)	15 (60.0)	NA
	3**	2 (6.3)	2 (8.0)	NA
	Intervention type in the experimental group, n (%)			
	Parenteral drugs	2 (6.3)	2 (8.0)	0
	Enteral drugs	10 (31.3)	7 (28.0)	3 (42.9)
	Topical drugs	3 (9.4)	3 (12.0)	0
	Interventional radiology	15 (46.9)	11 (44.0)	4 (57.1)
	Physiotherapy	2 (6.3)	2 (8.0)	0
	Control group treatment (n = 31)^a^, n (%)			
	Placebo, sham interventions	4 (12.5)	4 (16.0)	0
	Active drugs	5 (15.6)	5 (20.0)	0
	Interventional radiology	13 (40.6)	11 (44.0)	2 (28.6)
	Physiotherapy	0	0	0
	Surgery	1 (3.1)	0	1 (14.3)
	Interventional radiology and surgery	1 (3.1)	0	1 (14.3)
	No treatment	7 (21.9)	5 (20.0)	2 (28.6)

^a^One study did not have control group but had 2 experimental groups

* Whether the authors explained and/or justified the use of their study design/methodology in their protocol or publication

** For one, it was different concentrations of the same treatment and for the other, the groups were composed as follows: treatment 1 vs. treatment 2 vs. both treatments 1 and 2

**Table 3:** Characteristics of 11 comparative studies with a non-parallel design

	FIRST AUTHOR	DESIGN	YEAR	POPULATION	INTERVENTION	COMPARISON	PRIMARY OUTCOME
RANDOMIZED	Mükke	Cross-over [48]	2020	Venous malformation Adults and children	Physiotherapy (compression stockings)	No treatment	Change in volume of the malformation measured non-invasively by MRI.
	Novartis	Delayed-start [49]	2020	PROS Adults and children	Enteral drug (alpelisib)	Placebo	Change in the target lesion volumes assessed by MRI.
	Maruani	Randomized placebo phase [43]	2019	Lymphatic malformations Adults and children	Topical drug (sirolimus)	No treatment	Evaluation of global severity of the lingual microcystic lymphatic malformation using Physical Global Assessment (PGA).
	Leducq	Within-person [71]	2019	Lymphatic malformations Adults and children	Topical drug (sirolimus)	Placebo	Evaluation of cutaneous microcystic lymphatic malformation versus topical vehicle using PGA.
	Maruani	Randomized placebo phase [85]	2018	Vascular malformation Children	Enteral drug (sirolimus)	No treatment	Change in the volume of the vascular malformation seen on MRI.
	Smith	Delayed-start [103]	2009	Lymphatic malformations Children	Parenteral drug (picibanil)	No treatment	Change in volume/size of the malformation evaluated by CT or MRI.
NON-RANDOMIZED	te Loo	Challenge–dechallenge–rechallenge [74]	2019	Vascular malformation Adults and children	Enteral drug (sirolimus)	No treatment	Evaluation of the quality of life measured by TAPQOL, PedsQL and Research and development Rand-36. Evaluation of the pain using VAS score and NRS score.
	Parker	Observational run-in period [81]	2019	PROS Adults and children	Enteral drug (sirolimus)	No treatment	Percent change in unaffected and affected fibrofatty tissue measured by DXA and MRI scan.
	Hammer	Cross-over^a^ [65]	2018	Vascular malformation Adults and children	Enteral drug (sirolimus)	Patient’s long term history (Surgery, interventional radiology etc.)	Physical, functional, biological, radiological and quality of life evaluations.
	Weitz-Tuoretmaa	Historical control group [73]	2017	Venous malformation Adults and children	Sclerotherapy (polidocanol)	Sclerotherapy (ethanol)	Severity evaluation of symptoms by a self-evaluation questionnaire.
	James	Historical control group [66]	2011	Venous malformation Adults and children	Sclerotherapy	Surgery	Evaluation of lesions dimensions by MRI, surgical parameters, and duration of hospital stay.

^a^ According to the authors, this study is a “crossover trial using patient’s long-term clinical, biological and radiological history before sirolimus treatment as control”

Abbreviations: DXA, dual-energy X-ray absorptiometry; NRS, numerical rating scale; PedsQL, Pediatric Quality of Life Inventory; PROS, PIK3CA-related Overgrowth Spectrum; TAPQOL, TNO-AZL Preschool children Quality of Life; VAS, visual analogue scale

Among the 32 comparative studies, 14 (43.8%) had some form of blinding [43, 48, 49, 59, 67, 71, 80, 85, 86, 88, 91, 99, 103, 107]. These 14 studies were RCTs, representing 56% of RCTs included in the review. Blinded actors were outcome assessors (4 studies, 12.5%) [43, 85, 103, 107] or both participants and care providers (4 studies, 12.5%) [48, 71, 80, 86]. Three studies (9.4%) [49, 59, 99] exhibited triple blinding (participants, care provider and outcome assessor).

#### Design justification by the authors

For 6 of the 32 (18.8%) comparative studies, the authors justified their design in their report [43, 71, 73, 81, 85, 103]. Of these 6 studies, 4 were randomized [43, 71, 85, 103] and 2 were not [73, 81]. The first randomized study used a delayed-start design with an observational period of 6 months based on data suggesting that after this period, spontaneous regression was unlikely [103]. For the following 3 randomized studies [43, 71, 85], the authors explained that the population with the pathologies of interest was too rare to be able to set up a classic parallel-group trial. One used a within-person design because it allowed for reducing the number of patients to be included as well as inter-observation variability and all patients to receive the experimental treatment [71]. The other 2 randomized studies had a randomized placebo-phase design, which allowed for reducing the number of participants to include and increased acceptability (because every participant received the experimental intervention) and therefore ensured feasibility [43, 85]. For the non-randomized studies, the first used historical controls because randomization was judged too difficult to implement with the varying nature of VMs [73]. The second used an observational run-in period as a control and not a placebo because the experimental treatment was increasingly available and this would have compromised recruitment [81].

### Important changes in the protocol after trial beginning

After the beginning of the clinical trial, 10 (10.3%) studies made important changes to their protocol [7, 47, 63–65, 81, 87, 99, 110, 114]. Changes concerned the addition, removal or change of the primary or secondary outcomes (6 studies) [7, 47, 64, 65, 81, 87], a decrease in estimated enrollment (3 studies) [63, 99, 114], a modification of the intervention (1 study) [110] and a modification of the inclusion criteria (1 study) [47].

## Discussion

This methodological systematic literature search allowed identifying 62 reports and 35 ongoing studies representing 32 comparative and 65 non-comparative therapeutic studies of rare superficial VAs. Studies were frequently non-comparative cohorts, assessing interventional radiology in venous malformations or LMs. The proportion of experimental (i.e. randomized) studies was low. Our review showed that some authors used randomized designs distinct from the classical two-parallel group design, such as cross-over, within-person, randomized placebo-phase and delayed-start. We also noted in our review some designs of non-randomized studies that may be interesting for studying rare diseases such as the use of a historical control group or the challenge-dechallenge-rechallenge design [115].

This latter result is consistent with previously published results: in a systematic review of sirolimus treatment for LMs, among 20 studies, only one was an RCT, versus 19 retrospective case series or case reports [116]. Another review evaluating the efficacy of sirolimus for treating vascular abnormalities identified mostly single case reports (47 studies) and case series (22 studies) and very few prospective observational studies (n = 2) or RCTs (n = 2) [9]. Vascular anomalies are heterogeneous, which challenges the randomization (Is stratification desirable? possible?), outcome selection (Is there a relevant common outcome?) and results interpretation [9, 73, 117].

Thus, using a classical two parallel-group RCT presents a conundrum, and therefore, alternative designs involving intra-patient comparison are of high interest for clinical trials of rare pathologies because they can avoid some of the difficulties mentioned below [118–122]. The rarity of these pathologies is also a difficulty [116, 123]. Thus, we observed a lower number of included patients as compared with the planned sample size, a result already acknowledged for rare diseases [124, 125]. The most glaring example was one study that compared vincristine and sirolimus for treating high-risk VTs [63]. The authors had planned to enroll 50 patients, but the study had to stop because owing to the rarity of the pathology, only 4 patients had been recruited. Our observations seem consistent with the narrative review of Neto et al. which aimed to summarize Cochrane systematic review evidence on treatments for congenital vascular anomalies and hemangiomas. They had several difficulties including the limited number of existing systematic reviews, limited number of participants in the studies of these pathologies and heterogeneity of the participants [126]. To carry out therapeutic clinical trials on rare superficial VAs, the results of our review suggest to select a design that can limit the required sample size as much as possible. To have as low a required sample size as possible, investigators can promote intra-patient comparisons, thus increasing power. This option also allows for better accuracy because of the absence of inter-patient variability [127, 128]. In our review, we found three such designs: the cross-over design [129–131], the randomized placebo-phase design [132, 133] and the within-person design [134]. More so, the main advantage of this last design is that it eliminates confounding factors between the arms of the trial because the treatments to be compared are given at the same time and not successively as in the cross-over design or the randomized placebo-phase design. It is particularly suitable for dermatology (e.g., VAs) practice. However, the resulting problem is that these designs are more sensitive to dropouts and missing data because each participant is their own control; therefore, a dropout potentially affects both groups.

Another difficulty is using a placebo. First, a sham intervention for surgery or interventional radiology is difficult [135], in particular for the ethical aspect because this fictitious intervention can cause excessive risk for participants. In general, invasive procedures such as surgeries should normally be tested against standard medical treatment or no treatment [136]. Then, it is important to limit the time spent on placebo, Limiting the time spent on placebo facilitates recruitment. For VAs, given that there are standard treatments, patients and physicians would be reluctant to have a real placebo-only control group. Therefore, there are designs that can limit this period under placebo, such as the randomized placebo-phase design. Nevertheless, to ensure the validity of the results, an effective placebo-phase duration must be established: short enough to avoid changes over time and long enough for valid measurements [15]. However, there is the risk of participants dropping out if the placebo-controlled phase is too long. Second, for rare diseases with a potentially shortened lifespan, parents may be reluctant to have their children receive a placebo [81, 137]. To overcome this, investigators can maximize on-treatment participants by giving each patient the experimental treatment at some point (this is the case in the cross-over design) or by ensuring that all patients end the study being exposed to the experimental treatment, which offers the opportunity to pursue this treatment (if possible) outside of the study context (this is the case in the delayed-start design [138] and the randomized placebo-phase design). Either way, maximizing on-treatment participants facilitates recruitment and increase acceptance and accrual. These types of design can be of interest if conducting a two-parallel group RCT would be difficult or unacceptable, for instance when assessing an experimental treatment for an incurable or fatal disease or when the disease affects the more fragile pediatric population (which is the case with VAs).

For investigators, using an observational run-in period allows for collecting useful clinical data, especially in the case of rare diseases such as VAs, screening out ineligible or non-compliant participants, and establishing baseline observations. However, it also has disadvantages, such as affecting the external validity of the study by excluding patients and affecting the internal validity by exaggerating the intention-to-treat effect (e.g., if a run-in period is used to exclude participants who do not tolerate the treatment, then potentially there will be only a large number of good responders in the following phase, which can give a too optimistic view of the treatment effect) [139].

It is also possible to integrate an “internal” pilot study (pilot phase) into a clinical trial that allows for integrating the pilot participants into the definitive study and therefore not “exhausting” the stock of patients eligible for the study. This is interesting in the case of rare diseases such as VAs. These pilot phases built into the trial do not require additional time or funds [15].

Our review highlighted the use of a historical control. Despite certain advantages such as avoiding the problem of recruiting patients, especially for studying rare diseases such as VAs, this design has several disadvantages: confounding effects [140] (baseline patient characteristic differences between treatment arms can prevent highlighting the treatment effect), selection bias [141, 142] (whereby patients receiving the experimental treatment are selected from a pool of “good responders”, thus resulting in an overestimation of the treatment effect), performance, and detection bias [143, 144] (because there is no blinding). To overcome these, it would be interesting to use advanced methods to manage confusion in observational studies, such as stratifying patients on the estimated propensity scores during the analysis of observational data [142]. The Bayesian statistical approach could also be of interest, combined with any design, although we could not find it in our review. This approach uses data from previous studies to form a prior probability distribution for treatment effect, combined with current trial data for a posterior distribution, from which conclusions can be drawn [13].

This systematic review has several limitations. First, it certainly does not present all the studies published on the subject given the use of several filters: we included only studies published in the English language and since the year 2000. In addition, most authors did not justify their choice of methodological scheme in their publication, which limited our collection of information on this subject. In addition, registry ongoing studies contain more missing data than do published articles. We can also consider limitations in the method we chose to follow by not looking at other methodological issues such as sequence generation and allocation concealment, which are issues of importance in randomized trials but probably of lower importance than blinding [145, 146].

## Conclusions

Comparative studies are mandatory for assessing treatments or interventions, but RCTs are rare in these diseases. Classical two parallel-group designs are of limited use in rare pediatric diseases, notably with large between-patient variability. New designs, more adapted to this specific medical context, are emerging and can overcome the limitations of testing treatments in parallel groups. Their use is necessary for conducting trials with a high level of evidence.

### Electronic supplementary material

Below is the link to the electronic supplementary material.

Supplementary Material 1

## Data Availability

The dataset supporting the conclusions of this article is available in the opensource online data repository hosted at Mendeley: doi: 10.17632/n2vs8spdhz.1 [147].

## References

[1] Wassef M, Blei F, Adams D, Alomari A, Baselga E, Berenstein A (2015). Vascular anomalies classification: recommendations from the International Society for the study of vascular anomalies. Pediatrics.

[2] Gallant SC, Chewning RH, Orbach DB, Trenor CC, Cunningham MJ (2021). Contemporary Management of Vascular Anomalies of the Head and Neck—Part 1: vascular malformations: a review. JAMA Otolaryngol Neck Surg.

[3] Keppler-Noreuil KM, Rios JJ, Parker VER, Semple RK, Lindhurst MJ, Sapp JC (2015). PIK3CA-related overgrowth spectrum (PROS): diagnostic and testing eligibility criteria, differential diagnosis, and evaluation. Am J Med Genet A.

[4] Nguyen HL, Bonadurer GF, Tollefson MM (2018). Vascular malformations and health-related quality of life: a systematic review and Meta-analysis. JAMA Dermatol.

[5] European Commission. Rare diseases. Public Health - European Commission. 2016. https://ec.europa.eu/health/non_communicable_diseases/rare_diseases_en. Accessed 19 Nov 2021.

[6] Orphan Drug Act. Orphan Drug Act of 1983. Pub L. No. 97–414, 96 Stat. 2049. 1993.

[7] Adams DM, Trenor CC, Hammill AM, Vinks AA, Patel MN, Chaudry G (2016). Efficacy and safety of Sirolimus in the treatment of complicated vascular anomalies. Pediatrics.

[8] Cîrstoveanu C, Bizubac AM, Mustea C, Manolache Ștefan, Istrate-Bârzan A, Sfrijan D (2021). Antiproliferative therapy with sirolimus and propranolol for congenital vascular anomalies in newborns (case reports). Exp Ther Med.

[9] Freixo C, Ferreira V, Martins J, Almeida R, Caldeira D, Rosa M (2020). Efficacy and safety of sirolimus in the treatment of vascular anomalies: a systematic review. J Vasc Surg.

[10] Venot Q, Blanc T, Rabia SH, Berteloot L, Ladraa S, Duong J-P (2018). Targeted therapy in patients with PIK3CA-related overgrowth syndrome. Nature.

[11] Iacobas I, Adams DM, Pimpalwar S, Phung T, Blei F, Burrows P (2020). Multidisciplinary guidelines for initial evaluation of complicated lymphatic anomalies-expert opinion consensus. Pediatr Blood Cancer.

[12] Makhija LK, Bhattacharya S (2017). Management of vascular anomalies: review of institutional management algorithm. Indian J Plast Surg Off Publ Assoc Plast Surg India.

[13] Baiardi P, Giaquinto C, Girotto S, Manfredi C, Ceci A (2011). TEDDY Network of Excellence. Innovative study design for paediatric clinical trials. Eur J Clin Pharmacol.

[14] Jacqz-Aigrain E, Choonara I (2006). Paediatric clinical pharmacology.

[15] Nair B (2019). Clinical trial designs. Indian Dermatol Online J.

[16] Dekkers OM, Egger M, Altman DG, Vandenbroucke JP (2012). Distinguishing case series from cohort studies. Ann Intern Med.

[17] Mathes T, Pieper D (2017). Clarifying the distinction between case series and cohort studies in systematic reviews of comparative studies: potential impact on body of evidence and workload. BMC Med Res Methodol.

[18] Gulsen F, Cantasdemir M, Solak S, Gulsen G, Ozluk E, Numan F (2011). Percutaneous sclerotherapy of peripheral venous malformations in pediatric patients. Pediatr Surg Int.

[19] Mai HM, Zheng JW, Zhou Q, Yang XJ, Wang YA, Fan XD (2013). Intralesional injection of pingyangmycin is a safe and effective treatment for microcystic lymphatic malformations in the tongue. Phlebology.

[20] Ozeki M, Nozawa A, Yasue S, Endo S, Asada R, Hashimoto H (2019). The impact of sirolimus therapy on lesion size, clinical symptoms, and quality of life of patients with lymphatic anomalies. Orphanet J Rare Dis.

[21] Chaudhry IA, Al-Saikhan F, Al-Sheikh O, Al-Rashed W, Shamsi FA, Arat YO (2014). Intralesional OK-432 (Picibanil) sclerosing agent in the treatment of orbital lymphangiomas. Invest Ophthalmol Vis Sci.

[22] Bhatnagar S. Is bleomycin effective anti-angiogenic agent for vascular anomalies? Pediatr Blood Cancer. 2015;62 (Bhatnagar S.) Mumbai, India:S236–7.

[23] Claesson G, Kuylenstierna R (2002). OK-432 therapy for lymphatic malformation in 32 patients (28 children). Int J Pediatr Otorhinolaryngol.

[24] Won JH, Kim BM, Kim C-H, Park SW, Kim MD (2004). Percutaneous sclerotherapy of lymphangiomas with acetic acid. J Vasc Interv Radiol JVIR.

[25] Rautio R, Keski-Nisula L, Laranne J, Laasonen E (2003). Treatment of lymphangiomas with OK-432 (Picibanil). Cardiovasc Intervent Radiol.

[26] Wu HW, Wang X, Zheng JW, Zhao HG, Ge J, Zhang L et al. Treatment of deep-seated facial microcystic lymphatic malformations with intralesional injection of pingyangmycin. Med U S. 2016;95. 10.1097/MD.0000000000004790 PMC5402574 27631231

[27] Yue H, Qian J, Elner VM, Guo J, Yuan Y-F, Zhang R (2013). Treatment of orbital vascular malformations with intralesional injection of pingyangmycin. Br J Ophthalmol.

[28] Xue L, Wang XK, Tong S, Cheng MS, Xu DP. Effect of electrochemical treatment on high-flow venous malformations assisted by computer techniques. Int J Oral Maxillofac Surg., Wang L, Tong XK, Cheng S, Xu MS. D.P.) Department of Oral and Maxillofacial Surgery, School of Stomatology, China Medical University, Shenyang, Liaoning, China:296.

[29] Groot D, Rao J, Johnston P, Nakatsui T (2003). Algorithm for using a long-pulsed nd:YAG laser in the treatment of deep cutaneous vascular lesions. Dermatol Surg Off Publ Am Soc Dermatol Surg Al.

[30] Khandpur S, Sharma VK (2010). Utility of intralesional sclerotherapy with 3% sodium tetradecyl sulphate in cutaneous vascular malformations. Dermatol Surg Off Publ Am Soc Dermatol Surg Al.

[31] JPRN-UMIN000028905. A multicenter, phase 3 study assessing efficacy and safety of the Sirolimus in the Treatment of intractable lymphatic anomalies (SILA study). https://www.who-int-trial-search-Trial2-aspx-Trial-JPRN-UMIN-000028905. 2017.

[32] Yuan S-M, Hong Z-J, Jiang H-Q, Wang J, Hu X-B (2014). Intralesional copper wire retention and pingyangmycin injection: an effective combinational therapy for complex venous malformation in soft tissue. Phlebology.

[33] Lee H-J, Kim T-W, Kim J-M, Kim G-W, Ko H-C, Kim B-S (2017). Percutaneous sclerotherapy using bleomycin for the treatment of vascular malformations. Int J Dermatol.

[34] Chen W-L, Yang Z-H, Bai Z-B, Wang Y-Y, Huang Z-Q, Wang Y-J (2008). A pilot study on combination compartmentalisation and sclerotherapy for the treatment of massive venous malformations of the face and neck. J Plast Reconstr Aesthetic Surg JPRAS.

[35] Wu HW, Wang X, Zhang L, Zhao HG, Wang YA, Su LX (2016). Interferon-alpha therapy for refractory kaposiform hemangioendothelioma: a single-center experience. Sci Rep.

[36] Chen A-W, Liu Y-R, Li K, Zhang K, Wang T, Liu S-H (2015). Efficacy of sclerotherapy with radio-opaque foam guided by digital subtraction angiography for the treatment of complex venous malformations of the head and neck. Br J Oral Maxillofac Surg.

[37] Abdelaziz O, Hassan F, Elessawy K, Emad-Eldin S, Essawy RE (2019). Image-guided percutaneous bleomycin and Bevacizumab Sclerotherapy of Orbital Lymphatic Malformations in Children. Cardiovasc Intervent Radiol.

[38] Fujiwara H, Gobara H, Hiraki T, Iguchi T, Matsui Y, Sakurai J (2015). Cryoablation of vascular malformations: a phase I clinical trial. Cardiovasc Intervent Radiol.

[39] Raichura ND, Alam MS, Noronha VO, Mukherjee B (2017). A prospective study of the role of intralesional bleomycin in orbital lymphangioma. J AAPOS Off Publ Am Assoc Pediatr Ophthalmol Strabismus.

[40] Ratnavel GR (2011). Foam sclerotherapy in various vascular and lymphatic malformations. Indian J Dermatol Venereol Leprol.

[41] ClinicalTrials.gov Identifier NCT00462462. Systemic and Local Diffusion of Ethanol After Administration of Ethanol 96% Formulated in a Gel and Ethanol 98% Solution by the Percutaneous Route, in Patients With Congenital Venous Malformations:Pharmacokinetic, Pharmacodynamic and Clinical Study. National Library of Medicine (US). 2007. https://ClinicalTrials.gov/show/NCT00462462. Accessed 25 Jan 2021.

[42] Identifier UMIN000023437. Clinical study of combination local injection sclerotherapy of bleomycin and OK-432 for intractable lymphatic malformations. International Clinical Trials Registry Platform. 2016. https://trialsearch.who.int/Trial2.aspx?TrialID=UMIN000023437. Accessed 25 Jan 2021.

[43] ClinicalTrials.gov Identifier NCT04128722. TOPical Sirolimus in linGUal Microcystic Lymphatic Malformation -TOPGUN. National Library of Medicine (US). 2019. https://ClinicalTrials.gov/show/NCT04128722. Accessed 25 Jan 2021.

[44] Luo QF, Gan Y (2013). Pingyangmycin with triamcinolone acetonide effective for treatment of lymphatic malformations in the oral and maxillofacial region. J Craniomaxillofac Surg.

[45] Crisan BV, Baciut M, Baciut G, Campian RS, Crisan L (2010). Laser treatment in oral and maxillofacial hemangioma and vascular malformations. Timisoara Med J.

[46] Schumacher M, Dupuy P, Bartoli J-M, Ernemann U, Herbreteau D, Ghienne C (2011). Treatment of venous malformations: first experience with a new sclerosing agent–a multicenter study. Eur J Radiol.

[47] ClinicalTrials.gov Identifier NCT03188068. Study of Miransertib (MK-7075) in Participants With PIK3CA-related Overgrowth Spectrum and Proteus Syndrome (MOSAIC) (MK-7075-002) (MOSAIC). National Library of Medicine (US). 2017. https://ClinicalTrials.gov/show/NCT03188068. Accessed 25 Jan 2021.

[48] ClinicalTrials.gov Identifier NCT04637997. Influence of Flat-knitted Compression Stockings Class I and II on Venous Malformations. National Library of Medicine (US). 2020. https://ClinicalTrials.gov/show/NCT04637997. Accessed 25 Jan 2021.

[49] ClinicalTrials.gov Identifier NCT04589650. Study Assessing the Efficacy, Safety and PK of Alpelisib (BYL719) in Pediatric and Adult Patients With PIK3CA-related Overgrowth Spectrum. National Library of Medicine (US). 2020. https://ClinicalTrials.gov/show/NCT04589650. Accessed 25 Jan 2021.

[50] ClinicalTrials.gov Identifier NCT04104464. Patient Reported Outcomes for Vascular Malformations EmbolizatioN (PROVEN). National Library of Medicine (US). 2019. https://ClinicalTrials.gov/show/NCT04104464. Accessed 25 Jan 2021.

[51] Mukherjee B (2018). Ultrasound guided Intralesional Injection of Bleomycin for Orbital Lymphangioma- A prospective study. Invest Ophthalmol Vis Sci.

[52] Hassan Y, Osman AK, Altyeb A (2013). Noninvasive management of hemangioma and vascular malformation using intralesional bleomycin injection. Ann Plast Surg.

[53] Ali H, Saleh M, Mohammed W (2017). Efficacy and safety of duplex-guided polidocanol foam sclerotherapy for venous malformations. Int Angiol J Int Union Angiol.

[54] Dompmartin A, Blaizot X, Théron J, Hammer F, Chene Y, Labbé D (2011). Radio-opaque ethylcellulose-ethanol is a safe and efficient sclerosing agent for venous malformations. Eur Radiol.

[55] Jerjes W, Upile T, Hamdoon Z, Mosse CA, Akram S, Morley S (2011). Interstitial PDT for vascular anomalies. Lasers Surg Med.

[56] Bai N, Chen Y-Z, Fu Y-J, Wu P, Zhang W-N (2014). A clinical study of pingyangmycin sclerotherapy for venous malformation: an evaluation of 281 consecutive patients. J Clin Pharm Ther.

[57] Regmi D, Bista M, Shrestha S, Shrestha D, Mahato NB (2017). Comparative study on efficacy of intralesional bleomycin injection in head and neck lymphangioma and vascular malformation. J Clin Diagn Res.

[58] ClinicalTrials.gov Identifier NCT04172922. Topical Rapamycin/Sirolimus for Complicated Vascular Anomalies and Other Susceptible Lesions. National Library of Medicine (US). 2019. https://ClinicalTrials.gov/show/NCT04172922. Accessed 25 Jan 2021.

[59] ClinicalTrials.gov Identifier NCT01347294. Compare Two Different Sclerosing Agents in the Treatment of Venous Malformations. National Library of Medicine (US). 2011. https://ClinicalTrials.gov/show/NCT01347294. Accessed 25 Jan 2021.

[60] Harjai MM, Jha M (2012). Intralesional bleomycin and sodium tetradecyl sulphate for haemangiomas and lymphangiomas. Afr J Paediatr Surg AJPS.

[61] Meng J, Zhuang Q-W, Gu Q-P, Zhang J, Li Z-P, Si Y-M (2014). Digital subtraction angiography (DSA) guided sequential sclerotherapy for maxillofacial vein malformation. Eur Rev Med Pharmacol Sci.

[62] Andreisek G, Nanz D, Weishaupt D, Pfammatter T (2009). MR imaging-guided percutaneous sclerotherapy of peripheral venous malformations with a clinical 1.5-T unit: a pilot study. J Vasc Interv Radiol JVIR.

[63] ClinicalTrials.gov Identifier NCT02110069 H. A Study to Compare Vincristine to Sirolimus for Treatment of High Risk Vascular Tumors. National Library of Medicine (US). 2014. https://ClinicalTrials.gov/show/NCT02110069. Accessed 25 Jan 2021.

[64] ClinicalTrials.gov Identifier NCT02638389. Efficacy and Safety of Sirolimus in Vascular Anomalies That Are Refractory to Standard Care. National Library of Medicine (US). 2015. https://ClinicalTrials.gov/show/NCT02638389. Accessed 25 Jan 2021.

[65] Hammer J, Seront E, Duez S, Dupont S, Van Damme A, Schmitz S (2018). Sirolimus is efficacious in treatment for extensive and/or complex slow-flow vascular malformations: a monocentric prospective phase II study. Orphanet J Rare Dis.

[66] James CA, Braswell LE, Wright LB, Roberson PK, Moore MB, Waner M (2011). Preoperative sclerotherapy of facial venous malformations: impact on surgical parameters and long-term follow-up. J Vasc Interv Radiol JVIR.

[67] Johann ACBR, Aguiar MCF, do Carmo MAV, Gomez RS, Castro WH, Mesquita RA (2005). Sclerotherapy of benign oral vascular lesion with ethanolamine oleate: an open clinical trial with 30 lesions. Oral Surg Oral Med Oral Pathol Oral Radiol Endod.

[68] JPRN-UMIN000030522. A multicenter, single-arm, prospective study assessing efficacy and safety of the Sirolimus in the treatment of intractable vascular anomalies. 2017. https://www.who-int-trial-search-Trial2-aspx-TrialIDJPRN-UMIN000030522

[69] ClinicalTrials.gov Identifier NCT02042326 A. Prospective Evaluation of the Efficacy of Sirolimus (Rapamune®) in the Treatment of Severe Arteriovenous Malformations. National Library of Medicine (US). 2014. https://ClinicalTrials.gov/show/NCT02042326. Accessed 25 Jan 2021.

[70] ClinicalTrials.gov Identifier NCT04258046. Trametinib in the Treatment of Complicated Extracranial Arterial Venous Malformation. National Library of Medicine (US). 2020. https://ClinicalTrials.gov/show/NCT04258046. Accessed 25 Jan 2021.

[71] Leducq S, Caille A, Barbarot S, Bénéton N, Bessis D, Boccara O (2019). Topical sirolimus 0.1% for treating cutaneous microcystic lymphatic malformations in children and adults (TOPICAL): protocol for a multicenter phase 2, within-person, randomized, double-blind, vehicle-controlled clinical trial. Trials.

[72] Yamaki T, Nozaki M, Sakurai H, Takeuchi M, Soejima K, Kono T (2008). Prospective randomized efficacy of ultrasound-guided foam sclerotherapy compared with ultrasound-guided liquid sclerotherapy in the treatment of symptomatic venous malformations. J Vasc Surg.

[73] Weitz-Tuoretmaa A, Keski-Nisula L, Rautio R, Laranne J (2018). Quality of life after endovascular sclerotherapy of low-flow venous malformations: the efficacy of polidocanol compared with ethanol. Acta Radiol.

[74] ClinicalTrials.gov Identifier NCT0398715. Treatment of Congenital Vascular Malformations Using Sirolimus: Improving Quality of Life. National Library of Medicine (US). 2019. https://ClinicalTrials.gov/show/NCT03987152. Accessed 25 Jan 2021.

[75] Baskota DK, Singh BB, Sinha BK (2007). OK-432: an effective sclerosing agent for the treatment of lymphangiomas of head and neck. Kathmandu Univ Med J KUMJ.

[76] Lamba S, Keshava SKN, Moses V, Surendrababu N, Gupta AK (2012). Ethanol sclerotherapy for treatment of venous malformations of face and neck- A single centre experience. Eur J Plast Surg.

[77] ClinicalTrials.gov Identifier NCT01212965. Selenium in the Treatment of Complicated Lymphatic Malformations. National Library of Medicine (US). 2010. https://ClinicalTrials.gov/show/NCT01212965. Accessed 25 Jan 2021.

[78] Li J, Chen J, Zheng G, Liao G, Fu Z, Li J (2010). Digital subtraction angiography-guided percutaneous sclerotherapy of venous malformations with pingyangmycin and/or absolute ethanol in the maxillofacial region. J Oral Maxillofac Surg Off J Am Assoc Oral Maxillofac Surg.

[79] Teusch VI, Wohlgemuth WA, Hammer S, Piehler AP, Müller-Wille R, Goessmann H (2017). Ethanol-gel sclerotherapy of venous malformations: effectiveness and safety. AJR Am J Roentgenol.

[80] Helal HA, Mahmoud N (2020). Effect of foam and liquid bleomycin in the management of venous malformations in head and neck region: a comparative study. J Plast Reconstr Aesthet Surg.

[81] Parker VER, Keppler-Noreuil KM, Faivre L, Luu M, Oden NL, De Silva L (2019). Safety and efficacy of low-dose sirolimus in the PIK3CA-related overgrowth spectrum. Genet Med.

[82] ClinicalTrials.gov Identifier NCT03767660. Efficacy of Rapamycin (Sirolimus) in the Treatment of BRBNS, Hereditary or Sporadic Venous Malformation. National Library of Medicine (US). 2018. https://ClinicalTrials.gov/show/NCT03767660. Accessed 25 Jan 2021.

[83] Wang X, Meng J, Zhang J, Wu R, Gu J, Shao C, Han K (2016). Curative effects of RF combined with DSA-guided ethanol sclerotherapy in venous malformations. Exp Ther Med.

[84] Jain R, Bandhu S, Sawhney S, Mittal R (2002). Sonographically guided percutaneous sclerosis using 1% polidocanol in the treatment of vascular malformations. J Clin Ultrasound JCU.

[85] ClinicalTrials.gov Identifier NCT02509468. suPERficial Slow-flow Vascular malFORMations Treated With sirolimUS. National Library of Medicine (US). 2015. https://ClinicalTrials.gov/show/NCT02509468. Accessed 25 Jan 2021.

[86] ClinicalTrials.gov Identifier NCT04409145. First in Human Trial of Topical VT30 in Pts With Venous/Lymphatic Malformations Assoc With PIK3CA or TEK Gene Mutations. National Library of Medicine (US). 2020. https://ClinicalTrials.gov/show/NCT04409145. Accessed 25 Jan 2021.

[87] Cornelis FH, Labreze C, Pinsolle V, Le Bras Y, Castermans C, Bader C, Thiebaut R, Midy D, Grenier N. Percutaneous image-guided cryoablation as second-line therapy of soft-tissue venous vascular malformations of extremities: a prospective study of Safety and 6-Month Efficacy. Cardiovasc Intervent Radiol. 2017;:1–9. 10.1007/s00270-017-1636-y 28361195

[88] EudraCT Number 2009-009956-20. Prospective, randomized, controlled, mono-centric, two-armed, single-blinded pilot study for the treatment of venous and capillary vascular malformations by Indocyanine Green-augmented laser therapy. EU Clinical Trials Register. 2009. https://www.clinicaltrialsregister.eu/ctr-search/trial/2009-009956-20/DE. Accessed 25 Jan 2021.

[89] Zhang J, Li H-B, Zhou S-Y, Chen K-S, Niu C-Q, Tan X-Y, Jiang YZ, Lin Q-Q (2013). Comparison between absolute ethanol and bleomycin for the treatment of venous malformation in children. Exp Ther Med.

[90] ChiCTR-INR-16009651. High selective noninvasive laser in suppressing infantile venous malformations: a prospective, multi-centered, randomized controlled clinical trial. 2016. https://www.who-int-trial-search-Trial2-aspx-Trial-ChiCTR-INR-16009651

[91] Song D, Guo L, Sheng H, Li J, Wang L, Wu C, Wang C, Niu Y, Zeng Q (2020). DSA-guided percutaneous sclerotherapy for children with oropharyngeal low-flow venous malformation. Exp Ther Med.

[92] Ballah D, Zhu X, Edgar JC, Cahill AM (2011). The role of C-Arm computed tomography in the treatment of venous malformations and prediction of local skin complications. J Investig Med.

[93] Zhi K, Bai H, Zhao M, Ren W, Wen Y (2007). The role of intraleisonal pingyangmycin in the treatment of maxilla and facial venous malformation. J Xian Jiaotong Univ Med Sci.

[94] Gorst CM, Sophos S, Liew S (2011). The use of the long-pulsed nd:YAG laser for venous malformations in the pediatric population. Lasers Med Sci.

[95] Emran MA, Dubois J, Laberge L, Al-Jazaeri A, Bütter A, Yazbeck S (2006). Alcoholic solution of zein (Ethibloc) sclerotherapy for treatment of lymphangiomas in children. J Pediatr Surg.

[96] Wang S, Zhang J, Ge W, Liu Y, Guo Y, Liu Y (2017). Efficacy and safety of oral sildenafil in treatment of pediatric head and neck lymphatic malformations. Acta Otolaryngol (Stockh).

[97] Liu Z, Li J, Song D, Wang L, Wang C, Guo L (2020). Evaluation of curative effect of different concentration of tube defects in pingyang mycin local injection in treatment of children with maxillofacial region large cystic lymphatic malformation. Acta Med Mediterr.

[98] Kella N, Rathi PK, Sheikh U, Qureshi MA (2011). Our experience of bleomycin sclerotherapy for peripheral lymphangioma in children and review of the literature. Pak J Med Sci.

[99] ClinicalTrials.gov Identifier NCT02335242. Sildenafil for the Treatment of Lymphatic Malformations. 2015. https://ClinicalTrials.gov/show/NCT02335242. Accessed 25 Jan 2021.

[100] Pandey V, Tiwari P, Sharma SP, Kumar R, Panigrahi P, Singh OP (2019). Development of a biomarker of efficacy in second-line treatment for lymphangioma of the tongue: a pilot study. Br J Oral Maxillofac Surg.

[101] ClinicalTrials.gov Identifier NCT03243019. Efficacy of Rapamycin in the Treatment of Cervico-facial Lymphatic Malformations. National Library of Medicine (US). 2017. https://ClinicalTrials.gov/show/NCT03243019. Accessed 25 Jan 2021.

[102] Saddal NS, Sharif A, Ahmad S, Mirza F, Akhtar N, Anwar-Ul-Haq (2007). Intralesional bleomycin injection a primary therapy for peripheral lymphangiomas. Pak J Med Sci.

[103] Smith MC, Zimmerman MB, Burke DK, Bauman NM, Sato Y, Smith RJH (2009). Efficacy and safety of OK-432 immunotherapy of lymphatic malformations. Laryngoscope.

[104] Muhammad KS, Hashmi SA, Hussain M, Ahmad S, Gillani K (2020). Efficacy of Intralesional Bleomycin Injection Sclerotherapy in Macrocystic Lymphangioma in paediatric patients. J Ayub Med Coll Abbottabad JAMC.

[105] ClinicalTrials.gov Identifier NCT04409691. SCMC Trial on KHE With KMP (V. 2020). National Library of Medicine (US). 2020. https://ClinicalTrials.gov/show/NCT04409691. Accessed 25 Jan 2021.

[106] ClinicalTrials.gov Identifier NCT04056962. Tacrolimus for the Treatment of Superficial Kaposiform Hemangioendothelioma and Tufted Angioma. National Library of Medicine (US). 2019. https://ClinicalTrials.gov/show/NCT04056962. Accessed 25 Jan 2021.

[107] ClinicalTrials.gov Identifier NCT04448873. Guided Discontinuation Versus Maintenance Treatment of Sirolimus in Pediatric Patients With Kaposiform Hemangioendothelioma. National Library of Medicine (US). 2021. https://ClinicalTrials.gov/show/NCT04448873. Accessed 25 Jan 2021.

[108] ClinicalTrials.gov Identifier NCT04598204. Efficacy and Safety of Rapamycin to Complex Vascular Anomalies in Pediatric Patients. National Library of Medicine (US). 2020. https://ClinicalTrials.gov/show/NCT04598204. Accessed 25 Jan 2021.

[109] ClinicalTrials.gov Identifier NCT04077515. Safety and Efficacy of Low-dose Sirolimus to Kaposiform Hemangioendothelioma. National Library of Medicine (US). 2019. https://ClinicalTrials.gov/show/NCT04077515. Accessed 25 Jan 2021.

[110] ClinicalTrials.gov Identifier NCT03188068. Sirolimus Versus Sirolimus Plus Prednisolone for Kaposiform Hemangioendothelioma With Kasabach-Merritt Syndrome. National Library of Medicine (US). 2017. https://clinicaltrials.gov/show/NCT03188068. Accessed 25 Jan 2021.

[111] ClinicalTrials.gov Identifier NCT01677624, Multicenter A. Open-label Study for E7040 in Japanese Subjects With Hypervascular Tumor and Subjects With Arteriovenous Malformation. National Library of Medicine (US). 2012. https://ClinicalTrials.gov/show/NCT01677624. Accessed 25 Jan 2021.

[112] JPRN-UMIN000012564. Phase II trial of embolization with NBCA-lipiodol mixture (JIVROSG-0802). 2013. https://www.who-int-trial-search-Trial2-aspx-TrialIDJPRN-UMIN000012564

[113] Sanlialp I, Karnak I, Tanyel FC, Senocak ME, Büyükpamukçu N (2003). Sclerotherapy for lymphangioma in children. Int J Pediatr Otorhinolaryngol.

[114] ClinicalTrials.gov Identifier NCT03583307. Efficacy and Safety of Sirolimus to Vascular Anomalies. National Library of Medicine (US). 2018. https://ClinicalTrials.gov/show/NCT03583307. Accessed 25 Jan 2021.

[115] Harbers VEM, Zwerink LGJM, Rongen GA, Klein WM, van der Vleuten CJM, van Rijnsoever IMP (2023). Clinical differences in sirolimus treatment with low target levels between children and adults with vascular malformations - a nationwide trial. Clin Transl Sci.

[116] Wiegand S, Wichmann G, Dietz A (2018). Treatment of lymphatic malformations with the mTOR inhibitor sirolimus: a systematic review. Lymphat Res Biol.

[117] De Maria L, De Sanctis P, Balakrishnan K, Tollefson M, Brinjikji W (2020). Sclerotherapy for lymphatic malformations of head and neck: systematic review and meta-analysis. J Vasc Surg Venous Lymphat Disord.

[118] Blin O, Lefebvre M-N, Rascol O, Micallef J (2020). Orphan drug clinical development. Therapies.

[119] Kruizinga MD, Zuiker RGJA, Sali E, de Kam ML, Doll RJ, Groeneveld GJ (2020). Finding suitable clinical endpoints for a potential treatment of a Rare Genetic Disease: the case of ARID1B. Neurotherapeutics.

[120] Qiu T, Wang Y, Dabbous M, Hanna E, Han R, Liang S (2020). Current state of developing advanced therapies for rare diseases in the European Union. Expert Opin Orphan Drugs.

[121] Whicher D, Philbin S, Aronson N (2018). An overview of the impact of rare disease characteristics on research methodology. Orphanet J Rare Dis.

[122] Rath A, Salamon V, Peixoto S, Hivert V, Laville M, Segrestin B (2017). A systematic literature review of evidence-based clinical practice for rare diseases: what are the perceived and real barriers for improving the evidence and how can they be overcome?. Trials.

[123] Giugliani L, Vanzella C, Zambrano MB, Donis KC, Wallau TKW, Costa FM et al. da,. Clinical research challenges in rare genetic diseases in Brazil. Genet Mol Biol. 2019;42:305–11. 10.1590/1678-4685-GMB-2018-0174 PMC6687354 31170279

[124] Hee SW, Willis A, Tudur Smith C, Day S, Miller F, Madan J (2017). Does the low prevalence affect the sample size of interventional clinical trials of rare diseases? An analysis of data from the aggregate analysis of clinicaltrials.gov. Orphanet J Rare Dis.

[125] Hilgers R (2016). Design and analysis of clinical trials for small rare disease populations. J Rare Dis Res Treat.

[126] Guedes Neto HJ, Kuramoto DAB, Correia RM, Santos BC, Borges A, Pereda C (2022). What do Cochrane systematic reviews say about congenital vascular anomalies and hemangiomas? A narrative review. Sao Paulo Med J.

[127] Abrahamyan L, Feldman BM, Tomlinson G, Faughnan ME, Johnson SR, Diamond IR (2016). Alternative designs for clinical trials in rare diseases. Am J Med Genet C Semin Med Genet.

[128] Mitroiu M, Oude Rengerink K, Pontes C, Sancho A, Vives R, Pesiou S (2018). Applicability and added value of novel methods to improve drug development in rare diseases. Orphanet J Rare Dis.

[129] Hills M, Armitage P (1979). The two-period cross-over clinical trial. Br J Clin Pharmacol.

[130] Nguyen J, Takebe N, Kummar S, Razak A, Chawla SP, George S et al. Randomized Phase II Trial of Sunitinib or Cediranib in Alveolar Soft Part Sarcoma. Clin Cancer Res. 2022;OF1–9. 10.1158/1078-0432.CCR-22-2145 PMC10068440 36302173

[131] Thompson KP, Sykes J, Chandakkar P, Marambaud P, Vozoris NT, Marchuk DA (2022). Randomized, double-blind, placebo-controlled, crossover trial of oral doxycycline for epistaxis in hereditary hemorrhagic telangiectasia. Orphanet J Rare Dis.

[132] Feldman B, Wang E, Willan A, Szalai JP (2001). The randomized placebo-phase design for clinical trials. J Clin Epidemiol.

[133] Maruani A, Tavernier E, Boccara O, Mazereeuw-Hautier J, Leducq S, Bessis D (2021). Sirolimus (Rapamycin) for Slow-Flow Malformations in children: the observational-phase Randomized Clinical PERFORMUS Trial. JAMA Dermatol.

[134] Pandis N, Chung B, Scherer RW, Elbourne D, Altman DG (2017). CONSORT 2010 statement: extension checklist for reporting within person randomised trials. BMJ.

[135] Boutron I, Tubach F, Giraudeau B, Ravaud P (2004). Blinding was judged more difficult to achieve and maintain in nonpharmacologic than pharmacologic trials. J Clin Epidemiol.

[136] Miller F, Wendler D (2009). The ethics of sham invasive intervention trials. Clin Trials Lond Engl.

[137] Augustine EF, Adams HR, Mink JW (2013). Clinical trials in Rare Disease: Challenges and Opportunities. J Child Neurol.

[138] D’Agostino RB (2009). The delayed-start Study Design. N Engl J Med.

[139] Laursen DRT, Paludan-Müller AS, Hróbjartsson A (2019). Randomized clinical trials with run-in periods: frequency, characteristics and reporting. Clin Epidemiol.

[140] Mishra-Kalyani PS, Kordestani LA, Rivera DR, Singh H, Ibrahim A, DeClaro RA (2022). External control arms in oncology: current use and future directions. Ann Oncol.

[141] Papageorgiou SN, Koretsi V, Jäger A (2017). Bias from historical control groups used in orthodontic research: a meta-epidemiological study. Eur J Orthod.

[142] Ghadessi M, Tang R, Zhou J, Liu R, Wang C, Toyoizumi K (2020). A roadmap to using historical controls in clinical trials – by Drug Information Association Adaptive Design Scientific Working Group (DIA-ADSWG). Orphanet J Rare Dis.

[143] Moustgaard H, Clayton GL, Jones HE, Boutron I, Jørgensen L, Laursen DRT (2020). Impact of blinding on estimated treatment effects in randomised clinical trials: meta-epidemiological study. BMJ.

[144] Saltaji H, Armijo-Olivo S, Cummings GG, Amin M, da Costa BR, Flores-Mir C (2018). Influence of blinding on treatment effect size estimate in randomized controlled trials of oral health interventions. BMC Med Res Methodol.

[145] Rhodes KM, Turner RM, Savović J, Jones HE, Mawdsley D, Higgins JPT (2018). Between-trial heterogeneity in meta-analyses may be partially explained by reported design characteristics. J Clin Epidemiol.

[146] Moher D, Hopewell S, Schulz KF, Montori V, Gøtzsche PC, Devereaux PJ (2010). CONSORT 2010 explanation and elaboration: updated guidelines for reporting parallel group randomised trials. BMJ.

[147] Allemang-Trivalle A. Designs used in published therapeutic studies of rare superficial vascular anomalies: a systematic literature search. Mendeley Data. 2023;V1. 10.1186/s12874-023-02017-0 PMC10466846 37648985

