# Supplementary material for: Designs used in published therapeutic studies of rare superficial vascular anomalies: a systematic literature search
Source: BMC Med Res Methodol. 2023 Aug 30;23:196. doi: 10.1186/s12874-023-02017-0 (PMC10466846; doi:10.1186/s12874-023-02017-0)
Supplement: Supplementary file 1 — Supplementary Material 1 [file 12874_2023_2017_MOESM1_ESM.docx]

**SUPLEMENTARY MATERIAL**

**Journal name: BMC Medical Research Methodology**

**Designs used in published therapeutic studies of rare superficial vascular anomalies: A systematic literature search**

Aude Allemang-Trivalle^1,2^, Sophie Leducq^1,3^, Annabel Maruani^1, 2, 3*^, Bruno Giraudeau^1,2*^

^1^ Université de Tours, Université de Nantes, INSERM, SPHERE U1246, Tours, France

^2^ INSERM CIC1415, CHRU de Tours, Tours, France

^3^ Department of Dermatology, Reference Center for Genodermatoses and Rare Skin Diseases (Maladies Génétiques rares à Expression Cutanée–Tours), CHRU de Tours, Tours, France

* These two authors equally contributed

**Correspondence:** Aude Allemang-Trivalle, Centre Hospitalier Régional Universitaire Tours, Clinical Investigation Center 1415, 37044 Tours Cedex 9, France. E-mail: [aude.allemang-trivalle@etu.univ-tours.fr](mailto:aude.allemang-trivalle@etu.univ-tours.fr)

**SUPPLEMENTARY FIGURE**


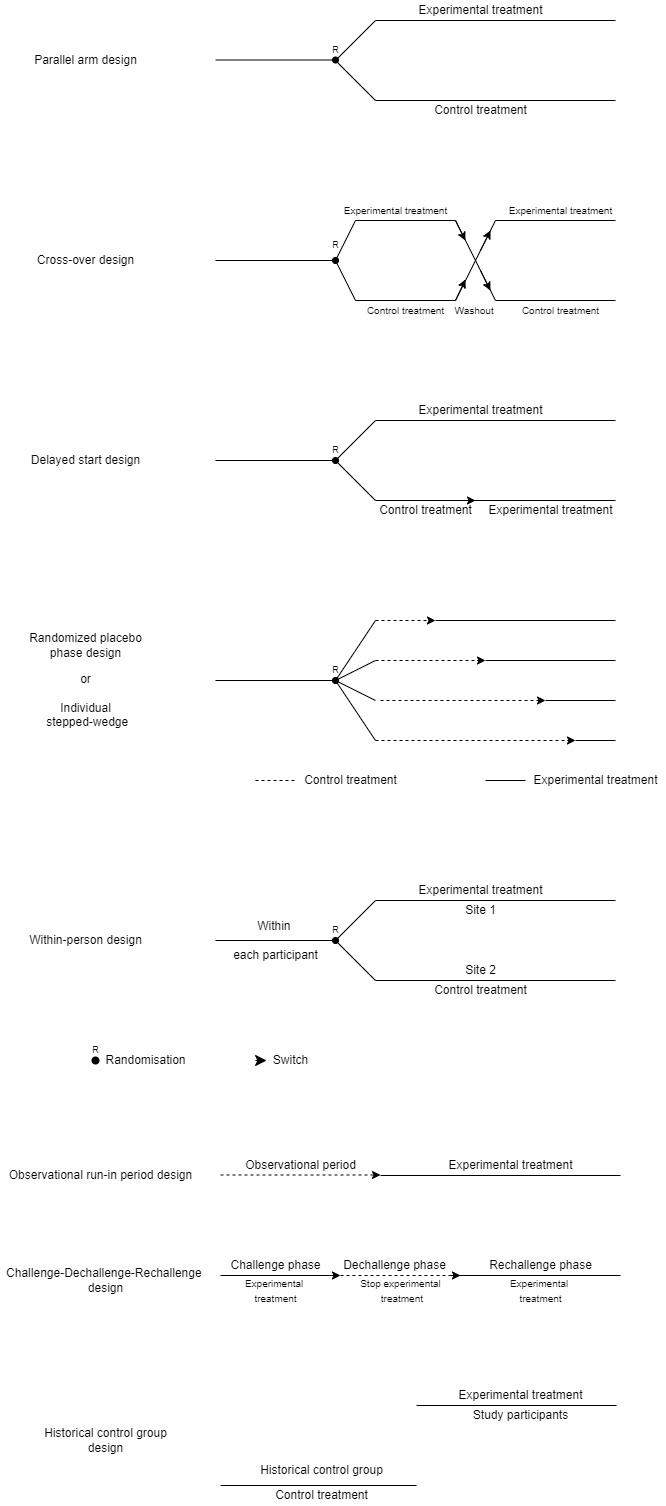


**Fig. S1 Designs used in comparative studies included in the final analysis** Parallel-group, cross-over, delayed-start, within-person and individual stepped-wedge designs are randomized designs. The observational run-in period, challenge–dechallenge–rechallenge and historical control group designs are not randomized. “R” refers to the randomization and the triangle to the switch between the control treatment and the experimental treatment or vice versa.

**SEARCH TERMS**

**Pubmed**

("Vascular anomal*" OR "Vascular malformation*" OR "Vascular tumor*" OR "Kasabach-Merritt" OR "Lymphangiom*" OR "Lymphatic malformation*" OR "Venous malformation*" OR "PIK3CA-related overgrowth spectrum" OR “Klippel-Trenaunay syndrome” OR “CLOVES”) AND (“Efficac*” OR "Trial*" OR "Random*” OR "Therapeutic stud*") NOT (Case report*[TI]) NOT(cancer*[TI]) NOT(retrospectiv*[TI])

**Central**

("Vascular anomal*" OR "Vascular malformation*" OR "Vascular tumor*" OR "Kasabach-Merritt" OR "Lymphangiom*" OR "Lymphatic malformation*" OR "Venous malformation*" OR "PIK3CA -related overgrowth spectrum" OR “Klippel-Trenaunay syndrome” OR “CLOVES”) in Title Abstract Keyword AND ("Efficac" OR "Trial*" OR "Random* " OR "Therapeutic*") in Title Abstract Keyword NOT "Cancer* ", "Case report*", "Retrospectiv*" in Record Title - (Word variations have been searched)

**Embase**

('vascular anomal*':ab,ti OR 'vascular malformation*':ab,ti OR 'vascular tumor*':ab,ti OR 'kasabach-merritt':ab,ti OR 'lymphangiom*':ab,ti OR 'lymphatic malformation*':ab,ti OR 'venous malformation*':ab,ti OR 'pik3ca-related overgrowth spectrum':ab,ti OR 'klippel-trenaunay syndrome':ab,ti OR 'cloves':ab,ti) AND ('efficac*':ab,ti OR 'trial*':ab,ti OR 'random*':ab,ti OR 'therapeutic stud*':ab,ti) NOT 'case report*':ti NOT cancer*:ti NOT retrospectiv*:ti AND [english]/lim AND [2000-2021]/py AND [humans]/lim AND [embase]/lim NOT [medline]/lim

**ClinicalTrials.gov**

Condition or disease: "Vascular anomalies" OR "Vascular malformations" OR "Vascular tumors" OR "Kasabach-Merritt" OR "Lymphangiomas" OR "Lymphatic malformations" OR "Venous malformations" OR "PIK3CA-related overgrowth spectrum" OR "Klippel-Trenaunay syndrome" OR "CLOVES"

Other therms: "Efficacy" OR "Trials" OR "Randomized" OR "Therapeutic studies" NOT "case reports" NOT "retrospective" NOT "cancers"

First posted: 01/01/2000 – 01/25/2021

**EU-CTR**

(CLOVES AND Efficacy) OR (CLOVES AND Trials) OR (CLOVES AND Randomized) OR (CLOVES AND Therapeutic studies) OR (Vascular anomalies AND Efficacy) OR (Vascular anomalies AND Trials) OR (Vascular anomalies AND Randomized) OR (Vascular anomalies AND Therapeutic studies) OR (Vascular malformations AND Efficacy) OR (Vascular malformations AND Trials) OR (Vascular malformations AND Randomized) OR (Vascular malformations AND Therapeutic studies) OR (Vascular tumors AND Efficacy) OR (Vascular tumors AND Trials) OR (Vascular tumors AND Randomized) OR (Vascular tumors AND Therapeutic studies) OR (Kasabach-Merritt AND Efficacy) OR (Kasabach-Merritt AND Trials) OR (Kasabach-Merritt AND Randomized) OR (Kasabach-Merritt AND Therapeutic studies) OR (Lymphangioma AND Efficacy) OR (Lymphangioma AND Trials) OR (Lymphangioma AND Randomized) OR (Lymphangioma AND Therapeutic studies) OR (Lymphatic malformations AND Efficacy) OR (Lymphatic malformations AND Randomized) OR (Lymphatic malformations AND Therapeutic studies) OR (Venous malformations AND Efficacy) OR (Venous malformations AND Trials) OR (Venous malformations AND Randomized) OR (Venous malformations AND Therapeutic studies) OR (Klippel-Trenaunay syndrome AND Efficacy) OR (Klippel-Trenaunay syndrome AND Trials) OR (Klippel-Trenaunay syndrome AND Randomized) OR (Klippel-Trenaunay syndrome AND Therapeutic studies) OR (PIK3CA-related overgrowth spectrum AND Efficacy) OR (PIK3CA-related overgrowth spectrum AND Trials) OR (PIK3CA-related overgrowth spectrum AND Randomized) OR (PIK3CA-related overgrowth spectrum AND Therapeutic studies) NOT(cancer) NOT(retrospective) NOT(case report)
